# Chemotherapy for hormone-resistant prostate cancer: Where are we today?

**DOI:** 10.4103/0970-1591.30269

**Published:** 2007

**Authors:** Tomas Buchler, Stephen J. Harland

**Affiliations:** Department of Oncology, University College London Hospital, London, United Kingdom

**Keywords:** Chemotherapy, docetaxel, estramustine, hormone-refractory prostate cancer, mitoxantrone

## Abstract

Significant progress has been achieved in chemotherapy for hormone-resistant prostate cancer (HRPC) in the last five years. Although the disease was long considered to be chemoresistant, docetaxel-based regimens in particular have been shown to both palliate symptoms and prolong survival in HRPC patients. Docetaxel is now considered the best available chemotherapy for prostate cancer progressing on first-line hormonal treatment. Other cytotoxics including mitoxantrone, anthracyclines, vinorelbin and vinblastine can alleviate symptoms and improve progression-free survival in HRPC without affecting overall survival. The survival benefit from chemotherapy seen in randomized studies has been small or nonexistent. Results of a recent trial suggest that the survival benefit may have been underestimated as a result of crossover from the less active to the active arm.

Chemotherapy has been used in hormone-resistant prostate cancer (HRPC) for more than two decades to palliate symptoms. Recently, two large studies have shown not only significant palliative benefits but also a small survival advantage for HRPC patients treated by combination chemotherapy based on docetaxel. In this review, we will focus on Phase III trials that have been published *in extenso* and that provide the current evidence base for the use of chemotherapy in HRPC.

Many cytotoxic drugs have shown some activity in advanced prostate cancer. The more commonly used ones include doxorubicin, mitoxantrone, epirubicin, cyclophosphamide, vinblastine, vinorelbin, cisplatin, taxanes and methotrexate. The rate of objective responses for these drugs in monotherapy was approximately 10 to 20%.[1] However, the evaluation of responses in these early trials was based on relatively insensitive, if important, outcomes, such as decrease in pain or decrease in the volume of measurable disease which is present only in a minority of patients.

The routine availability of prostate-specific antigen (PSA) testing since the late 1980s has enabled more refined designs and evaluation of clinical trials. Also, for the first time, it enabled trials to be aimed at asymptomatic HRPC patients. A reduction in PSA is often used as a surrogate endpoint and there is evidence that posttherapy PSA decline of more than 50% lasting for at least 28 days correlates with prolonged overall and progression-free survival in HRPC.[2] Whilst this is not an irrefutable proof of its clinical value, PSA reduction ≥ 50% has achieved widespread acceptance as an endpoint in clinical studies.[3] Recent data from trials of docetaxel-based regimes have renewed the discussion about the correlation between PSA response and prolonged survival. In an analysis of the data from the Southwest Oncology Group (SWOG) 99-16 randomized trial, Petrylak *et al* reported that a decline of PSA by 30% or more maintained over at least three months was associated with improved survival even after adjusting for treatment.[4] Prostate-specific antigen doubling time is another parameter that might be a valid prognostic marker for the outcome of HRPC treatment with modern chemotherapy.[5]

Androgen receptor signaling still plays an important role even in the hormone-refractory phase of prostate cancer and it is recommended that GnRH agonists be continued throughout chemotherapy to block the pathway.[6,7] Where endocrine therapy is temporarily discontinued, dramatic surges in PSA are seen.[8] In recent trials, cytotoxic drugs are usually combined with corticosteroids which suppress adrenal production of androgens but possibly also have a direct effect on the malignant cells. As responses to corticosteroid therapy alone are common, uncontrolled studies that incorporate a corticosteroid - even if only administered as antiemetics or for the prophylaxis of a hypersensitivity reaction - should be regarded with caution.

## MITOXANTRONE

Mitoxantrone has been the mainstay of chemotherapy for HRPC since the palliative benefit of the treatment was reported by Tannock *et al* in 1996.[9] In his important study, mitoxantrone 12 mg/m^2^ in 21-day intervals to a cumulative dose of 140 mg/m^2^ plus prednisone 5 mg twice daily (MP group) was compared to prednisone alone. As this was a study of palliation, patients with ECOG performance status ≤ 3 were included. The primary endpoint was a 2-point reduction in a 6-point pain intensity scale and this was achieved in 29% of patients in the MP group versus 12% of patients in the prednisone group (*P*=0.01). Among the 34 patients who received a cumulative dose of mitoxantrone higher than 100 mg/m^2^, there were five cases of presumed mitoxantrone-induced cardiac abnormalities. No statistically significant differences between the study arms in overall survival or PSA responses were detected. There was a significant crossover rate (50 of 81 patients in the prednisone group; 62%) which may account for the lack of survival difference [Table 1].[9]

**Table 1:** Results of Phase III chemotherapy trials using mitoxantrone-based regimens for hormone-resistant prostate cancer

Reference	No. patients	Treatment arm	Median survival (months)	Median time to progression (months)	Prostate-specific antigen (months) decrease ≥ 50% (%)
Tannock 1996	161	mitoxantrone + prednisone	11	10*	33
		prednisone	11	4.5*	22
Kantoff 1999	242	mitoxantrone + hydrocortisone	12.3	3.7*	38*
		hydrocortisone	12.6	2.3*	22*
Berry 2002	120	mitoxantrone + prednisone	23	8.1*	48*
		prednisone	19	4.1*	24*

Statistically significant differences are marked with an asterisk

The analgesic benefit of mitoxantrone and the lack of improvement in survival were confirmed in a subsequent controlled randomized study by Kantoff and collaborators who randomized 242 patients to mitoxantrone 14 mg/m^2^ every three weeks with hydrocortisone 40 mg daily or to hydrocortisone 40 mg only [Table 1].[10] In this study, patients in the hydrocortisone arm were not allowed to cross over to mitoxantrone or doxorubicin but other chemotherapy regimens were allowed in both treatment arms after progression or treatment failure.[10]

The role of mitoxantrone in asymptomatic men with HRPC was studied by Berry *et al*.[11] They allocated 120 patients to either six three-weekly cycles of mitoxantrone 12 mg/m^2^ with prednisone 10 mg daily or to prednisone 10 mg daily only. These patients had good performance status (ECOG 0 or 1 in 119 of 120 patients). Significantly better PSA responses and improved time to progression were seen in the mitoxantrone group [Table 1]. The overall survival (OS) was not affected. The crossover to mitoxantrone in the prednisone group was substantial but not quantified by the authors.[11]

As shown in Table 1, in these Phase III trials, mitoxantrone-based regimens proved effective for palliation of pain with responses in about 30% of patients with HRPC with reasonable toxicity. The duration of these palliative responses was about 10 months.[9] Significant crossover rates to chemotherapy in the control groups may be responsible for the lack of detected difference in OS. The inability of mitoxantrone to prolong life in men with HRPC in these studies prompted clinical trials using newer drugs with activity shown in Phase II studies.

## TAXANES

Earlier studies of paclitaxel and docetaxel showed promising activity in HRPC both as monotherapy and in combinations with drugs such as estramustine and/or glucocorticosteroids. In Phase I and Phase II studies of docetaxel and paclitaxel, PSA responses were achieved in approximately 50% of patients.[12] Some studies of the docetaxel-estramustine combination reported PSA responses in up to 68% of patients.[13] The principal toxicities were myelosuppression and fatigue.[12]

Recently, two multicentric randomized Phase III studies sponsored by the manufacturer of docetaxel have compared docetaxel-based chemotherapy with a mitoxantrone.

The Southwest Oncology Group (SWOG) 99-16 trial randomized 770 men who progressed after androgen ablation and antiandrogen withdrawal to treatment with docetaxel plus estramustine (DE) or mitoxantrone plus prednisone (MP).[14] Estramustine is a conjugate of an estrogen and the alkylating agent chlormethin. It is thought to act on malignant cells via hormonal mechanism and inhibition of microtubular function.[15] Its alkylating properties seem to be responsible for its toxicity. Estramustine was shown to have a synergistic effect with taxanes *in vitro*[16,17] and the combination appeared promising in small clinical trials.[18–20]

The DE group of the SWOG 99-16 trial was treated with 60 mg/m^2^ of docetaxel (increased to 70 mg/m^2^ in further cycles if no toxicity was seen) on Day 2 and estramustine 280 mg three times daily on Days 1 to 5 in a 21-day cycle. Dexamethasone 60 mg was given prior to docetaxel infusion. Most of these patients also received low-dose warfarin and aspirin to counter the prothrombotic effects of estramustine. The MP group was treated by mitoxantrone 12 mg/m^2^ (increased to 14 mg/m^2^ in later cycles if there was no toxicity) on Day 1 and prednisone 5 mg twice daily in a 21-day cycle. A maximum of 12 cycles of the DE combination was given. The cumulative dose of mitoxantrone was not to exceed 144 mg/m^2^. Severe toxicities were relatively uncommon in both arms. There was a significant difference in the occurrence of neutropenic fever (5 to 2% for DE and MP, respectively) as well as nausea and vomiting and cardiovascular events which were all more common in the DE group. Twelve treatment-related deaths were reported, eight in the DE group and four in the MP group. About half of the patients in both groups crossed over to other treatment(s) upon progression. Patients allocated to docetaxel and estramustine had longer median survival (17.5 months versus 15.6 months for mitoxantrone and prednisone; *P* = 0.02) [Table 2]. The PSA responses were also more common in the DE group than in the MP group (*P*<0.001). No differences were detected between the study groups in partial responses as defined by decrease in measurable disease or in pain palliation.[14,21]

**Table 2:** Results of randomized trials using docetaxel-based regimens for hormone-resistant prostate cancer

Reference	No. patients	Treatment arm	Median survival (months)	Median time to progression (months)	Prostate-specific antigen decrease ≥ 50% (%)
Petrylak 2004	770	docetaxel + estramustine	17.5*	6.3*	50*
		mitoxantrone + prednisone	15.6*	3.2*	27*
Tannock 2004	1006	docetaxel (3-weekly) + prednisone	18.9*	n.e.	45*
		docetaxel (weekly) + prednisone	17.4		48*
		mitoxantrone+prednisone	16.5*		32*
Oudard 2005	127	docetaxel (3-weekly) + estramustine + prednisone	18.6*	8.8*	67*
		docetaxel (Days 2 and 9) + estramustine + prednisone	18.4*	9.3*	63*
		mitoxantrone + prednisone	13.4*	1.7*	18*
Fossa 2006	134	docetaxel + prednisone	27*	11*	65*
		prednisone	18*	3*	34*

Statistically significant differences are marked with an asterisk

Tannock *et al* performed a randomized, multicentric clinical study comparing two different docetaxel regimens with mitoxantrone and prednisone.[22] The TAX 327 trial enrolled 1006 men with HRPC. Patients allocated to docetaxel-based arms received docetaxel 75 mg/m^2^ three-weekly (D3WP) or docetaxel 30 mg/m^2^ weekly for five weeks in a six-week cycle (D1WP). These patients also received dexamethasone 24 mg as a premedication before docetaxel. The patients in the mitoxantrone arm (MP) were treated by mitoxantrone 12 mg/m^2^ on Day 1 of a 21-day cycle. In addition, all study regimens included prednisone 10mg daily divided in two doses. Patients who received docetaxel had significantly more alopecia, diarrhea, stomatitis, tearing, dyspnea, peripheral edemas, epistaxis and Grade 3 or 4 neutropenia. Patients in the mitoxantrone group had higher occurrence of left ventricular dysfunction. Median survival for the D3WP group was 18.9 months versus 16.5 months for the MP group (*P*=0.009). The differences in median survival between the two docetaxel groups or between the D1WP group (17.4. months) and the MP group were not statistically significant. The PSA responses were significantly more common in the docetaxel groups (D3WP 45%, D1WP 48%, MP 32%) [Table 2]. Quality of life as measured by the FACT-P questionnaire was better for the docetaxel groups as compared to the mitoxantrone group despite the greater toxicity. Pain relief was better in the D3WP group (35%) as compared to the MP group (22%; *P*=0.01). The crossover rate in this trial was 20–27% for different study arms.[22]

A meta-analysis of trials of docetaxel-based regimens was presented recently by Oudard *et al*.[23] They pooled the data from the SWOG trial 99-16 and the TAX 327 together with their own data published in 2005 [Table 2].[24] The meta-analysis confirmed that docetaxel-based regimes significantly reduced the risk of death by 21% at 12 months and that the benefit persists in the long term, with the risk of death reduced by 8% at three years after the start of treatment.[23]

Taxanes are always administered with corticosteroid premedication (usually dexamethasone) and thus patients on the taxane arms received higher total dose of corticosteroids than the control groups. It is unclear whether this additional dose of corticosteroids contributed to positive results of taxane-based chemotherapy.

Perhaps a truer estimate of the survival benefit of taxane chemotherapy derives from an interesting trial recently reported by Fossa *et al*.[25] In a randomized Phase II trial they allocated 134 men to docetaxel (30mg/m^2^ weekly for five of every six weeks) plus prednisone 10mg daily or to prednisone alone. No crossover was allowed in the trial because in Norway chemotherapy was never given to HRPC patients. Progression-free survival (PFS) and PSA responses were also significantly better for patients receiving docetaxel (11 months and 65%, respectively) than for the control group (three months and 34%, respectively). Thus, the overall survival for the docetaxel arm was nine months longer than for the prednisone arm (27 months versus 18 months; *P*=0.02). The lack of crossover probably accounts for the superior results of chemotherapy with the prolongation of median OS by nine months in this study compared to only two months in the previously mentioned studies [Table 2].[26]

Despite the survival advantage, the responses in measurable disease were fairly low in the docetaxel studies, ranging from 8–17% of evaluable patients.[14,22] Thus, only a minority of patients with a PSA fall of more than 50% also had a partial response in measurable disease.

These studies have established docetaxel-based chemotherapy as a new standard in the treatment of HRPC. There is now evidence that regimens with docetaxel are the only therapy that provides not only palliative benefits but also a modest survival advantage in HRPC. Patients with SWOG performance status of ≤ 2 and elderly patients benefited from docetaxel-based chemotherapy as much as patients with better PS. Therefore these results are encouraging for patients with poor performance status who have traditionally only received supportive treatment might still benefit from chemotherapy.

## OTHER CHEMOTHERAPY REGIMENS

Several other regimens have been tested in Phase III trials. Invariably, they did prove some activity and improved PFS in HRPC but did not prolong overall survival.

Doxorubicin has been studied in HRPC in combination with prednisone,[26] with dose-escalated cyclophosphamide,[27] as well as in more complex chemohormonal regimens, such as doxorubicin with ketoconazole alternating with vinblastine plus estramustine.[28] The PSA response rate in these small trials ranged from 16–67%. The treatment with 20 mg/m^2^ of doxorubicin weekly in combination with prednisone 10 mg daily achieved symptom relief in approximately 50% of patients with no effect on survival.[26] In a randomized study comparing doxorubicin to 5-fluorouracil in 99 patients, doxorubicin was superior in terms of responses in measurable disease (25% vs. 8%; *P* ≤ 0.05) and in OS (29 vs. 24 weeks; *P* ≤ 0.05).[28] Other anthracyclines, including idarubicin and epirubicin have activity similar to doxorubicin in HRPC.[1] However, these drugs are used less commonly since mitoxantrone has received more attention in Phase III trials thanks to its relatively favorable toxicity profile.

Hudes *et al* studied estramustine 600 mg/m^2^ daily on days 1 to 42 of an eight-week cycle in combination with another antimicrotubule drug, vinblastine, given weekly in a dose of 4 mg/m^2^ for six consecutive weeks in a eight-week cycle.[29] The control arm received vinblastine alone according to the same schedule. While vinblastine has little effect in HRPC, it was hoped that the combination might be synergistic. The Phase III trial had overall survival as the primary endpoint. However, the difference in overall survival between the two groups was not statistically significant. In secondary endpoints, the estramustine-vinblastine combination proved to be superior in progression-free survival and PSA responses [Table 3]. Interestingly, the myelotoxicity of the estramustine-vinblastine combination was lower that that of vinblastine alone.[29]

**Table 3:** Results of Phase III chemotherapy trials using regimens based on tubule inhibitors for hormone-resistant prostate cancer

Reference	No. patients	Treatment arm	Median survival (months)	Median time to progression (months)	Prostate-specific antigen decrease ≥ 50% (%)
Hudes 1999	161	estramustine + vinblastine	11.9	3.7*	25*
		vinblastine	9.2	2.2*	3.2*
Abratt 2004	242	vinorelbine + hydrocortisone	24	3.7	30.1*
		hydrocortisone	24.6	2.8	19.2*

Statistically significant differences are marked with an asterisk

Abratt *et al* performed a randomized Phase III trial comparing another vinca alkaloid vinorelbine with hydrocortisone to hydrocortisone alone.[30] The dose of vinorelbine was 30 mg/m^2^ on days 1 and 8 of a three-week cycle and the dose of hydrocortisone in both study arms was 40 mg daily. Median PFS was marginally better in the vinorelbine group and median OS, not a primary objective in this study, was similar for both arms [Table 3]. The most common toxicity of vinorelbine was neutropenia and 26% of patients had a Grade 3 or 4 event.[30]

Satraplatin, an oral platinum drug was tested in an EORTC Phase III trial of satraplatin plus prednisone versus prednisone alone. The trial was closed recently after enrolling only 50 patients because the study drug was deemed to be of low commercial priority by the sponsor. Despite longer time to progression in the subgroup receiving satraplatin, differences in OS were not observed.[31] Satraplatin is being studied in several other Phase III trials for HRPC and the results are awaited in the near future.

Combination chemotherapy regimens that do not include hormonal agents have yet to be tested in Phase III trials. In our institution, we have extensive experience with the ECarboF regimen (epirubicin 50 mg/m^2^ and carboplatin AUC5 four-weekly and 5-fluorouracil 450 mg/m^2^sub with folinic acid two-weekly). This type of chemotherapy is well-tolerated and produces PSA and/or radiological responses in 45% of patients, with median time to progression of 9.2 months in patients younger than 70 years. This response rate is comparable to that achieved with docetaxel and corticosteroids are mostly avoided.[32]

## SECOND-LINE TREATMENTS FOR HRPC

One major unresolved issue is the management of HRPC progressing after first-line chemotherapy. The possibility of second-line chemotherapy with either docetaxel or mitoxantrone has been recently studied in several Phase II trials.[33] The largest of crossover trials comparing docetaxel and mitoxantrone as second-line agents was published by Michels *et al*.[34] These authors found that second-line docetaxel after first-line mitoxantrone-based chemotherapy produced better PSA responses than second-line mitoxantrone after docetaxel. Despite the PSA responses, neither of these strategies improved PFS or OS, which were two to three months and seven to 12 months, respectively. Moreover, 46% of patients in the mitoxantrone-after-docetaxel group and 64% patients in the docetaxel-after-mitoxantrone group had to have dose reduction, delay or cessation of chemotherapy or hospitalization as a result of toxicity.[34] Other studies confirmed the activity of taxane-based chemotherapy before or after mitoxantrone but the duration of responses is short and there is no difference in OS or PFS regardless of the drug sequence used.[35]

There are other, nonchemotherapy options for patients with HRPC who progress after chemotherapy. Glucocorticosteroids may be beneficial in the few patients who did not receive them as part of first-line chemotherapy. Ketoconazole may produce transient PSA responses but has not been evaluated extensively in this setting. Diethylstilboestrol, an estrogen, can induce PSA responses and pain relief and is our second-line treatment of choice.

The radionuclides strontium-89 and samarium-159 have been evaluated in Phase III trials with palliative endpoints in HRPC.[36–38] Both agents are, however, associated with long-term bone marrow suppression which makes them a problematic choice for those heavily pretreated and often elderly patients with significant comorbidities.

## UNRESOLVED ISSUES AND FUTURE DIRECTIONS

Docetaxel is currently the best available chemotherapy option for HRPC. Now that there is clear evidence for the potential of docetaxel regimens to prolong survival they have become an attractive option for patients with HRPC and their physicians. The cost of the treatment will remain a significant factor in the decision-making in the foreseeable future. In the UK, chemotherapy with docetaxel is about ten times as expensive as treatment with mitoxantrone, not taking into account additional costs as a result of higher toxicity rates. It has not been analyzed whether savings due to palliative benefits of docetaxel will at least partially compensate for the high cost of treatment.

It is not known currently whether chemotherapy has a role in the management of patients who are asymptomatic and only have biochemical relapse manifesting as rising PSA levels or whether it should be deferred until symptoms develop. The important question whether chemotherapy or second-line hormonal therapy is the optimal treatment of HRPC remains unanswered and clinical trials directly comparing the two modalities are lacking.

The potential of chemotherapy to improve long-term survival for hormone-sensitive prostate cancer is currently the subject of several Phase II trials where various regimens are administered as a part of initial treatment for locally advanced or metastatic disease. The combinations often include estramustine which has estrogenic activity which could account for a significant part of clinical responses.[39,40] Randomized studies are ongoing.

Perhaps the most important aspect of the Phase III trials testing docetaxel-based regimens is that they have proved the principle that survival in HRPC can be prolonged by chemotherapy. Several new cytotoxic drugs including epothilones and biologicals such as vaccines and angiogenesis inhibitors including atrasentan, thalidomide and bevacizumab, are currently being studied in Phase II trials for HRPC. Data will be available soon on the efficacy of these agents alone and in combination with docetaxel and it is possible that the outlook towards HRPC patients will be further improved by some of these promising novel agents.
